# Development of a peptide-based fluorescent probe for biological heme monitoring†
†Electronic supplementary information (ESI) available: Experimental details, HPLC and mass spectra for all peptides, UV-Vis spectra of peptides **AP3** and **CP3** with hemin. See DOI: 10.1039/c8ob02290a


**DOI:** 10.1039/c8ob02290a

**Published:** 2018-12-18

**Authors:** Laura D. Newton, Sofia I. Pascu, Rex M. Tyrrell, Ian M. Eggleston

**Affiliations:** a Department of Pharmacy and Pharmacology , University of Bath , Bath BA2 7AY , UK . Email: ie203@bath.ac.uk; b Department of Chemistry , University of Bath , Bath BA2 7AY , UK

## Abstract

Heme plays a vital role in cell biology and dysregulation of heme levels is implicated in a wide range of diseases. However, monitoring heme levels in biological systems is currently not straightforward. A short synthetic peptide probe containing 7-azatryptophan is shown to bind hemin *in vitro* with quenching of the azatryptophan fluorescence. This chemical tool can be used to detect the change in free heme induced in human skin cells upon exposure to UVA irradiation.

## Abstract 2

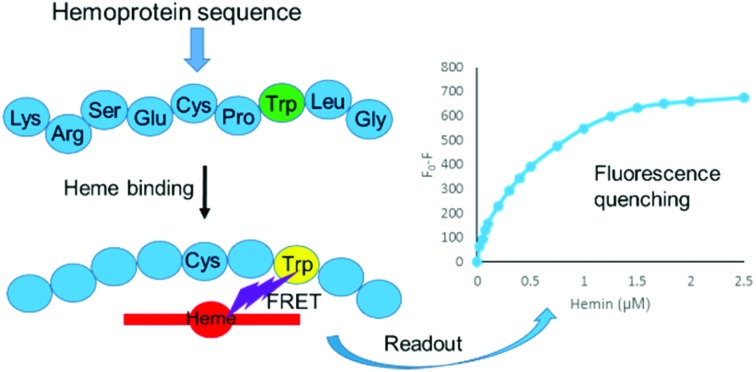
A prototype peptide-based probe has been developed for the determination of intracellular heme levels.

## 

Heme is an essential molecule for all aerobic organisms and is a prosthetic group for a large variety of proteins. While the generally accepted function of heme in eukaryotes is as a protein cofactor, it is now understood that free or labile heme is also an important cellular signalling molecule.^1^,^2^ The total heme content of the cell comprises the mostly inert heme that is tightly associated with hemoproteins such as cytochromes and the free heme pool which is available for regulatory protein binding and signalling. The protein-bound fraction is much larger than the free heme fraction, and represents the majority of cellular heme. Free heme can be toxic to the cell, catalysing the production of reactive oxygen species, promoting oxidative stress and inflammation.^3^,^4^ As a result the free heme pool is kept small and is therefore difficult to investigate and its role is much less well understood than that of heme in hemoproteins.^5^ Moreover, its dynamic mobilisation is required for all heme-dependent processes. Questions remain over the concentration of the free heme pool, cellular distribution, oxidation state and dynamics – how it is controlled and how it responds to different stimuli.

Organisms control their heme levels by a concerted action of several different systems: heme synthesis and degradation, import and export, and sequestration and scavenging by proteins.^4^ 5-Aminolevulinic acid synthase-1 (ALAS1) catalyses the first step of heme biosynthesis and its expression is negatively regulated by heme.^6^ Heme oxygenase-1 (HO-1) catabolises heme into carbon monoxide, iron and biliverdin and its expression is also regulated by heme. The transcription factor Bach1 acts as a negative regulator of the HO-1 gene but on heme binding, releases from the HO-1 promoter, allowing transcription.^7^,^8^

Dysregulation of heme levels has been implicated in a number of different diseases ranging from neurodegenerative disease^9^–^11^ and cancers^12^–^15^ to cardiac disease^16^–^18^ and diabetes.^19^,^20^ However, there are very few tools available that permit the study of the dynamics of free heme in live cells and this hampers investigation into the role of heme in disease.^5^ Cellular heme levels have been determined in the past by indirect spectroscopic methods which would typically involve homogenising cell or tissue samples and removing the central iron of heme from the porphyrin ring.^21^,^22^ These methods give one bulk measurement of the heme level of a population of cells, without distinguishing between the free and bound heme pool or the subcellular distribution of heme. An ideal heme probe would allow detection of heme in live cells in a manner that does not disrupt the physiology of the cell, and in this context fluorescence-based probes provide the potential to quantify heme levels in real time and on a cell-by cell basis.

A number of protein-based systems have been described for the determination of heme in biological systems based upon a fluorescence readout.^23^,^24^ These range from the use of fluorescently labelled HO-1 as a heme probe that functions *via* direct fluorescence quenching upon heme binding to the protein,^25^,^26^ to more sophisticated genetically encoded FRET probes that have been used to investigate the free heme pool.^27^–^29^ For example, the probe of Song *et al.*^27^ uses two heme-binding domains (IsdX1 and IsdC) that are each attached to a fluorescent protein (enhanced cyan fluorescent protein (ECFP) and enhanced yellow fluorescent protein (EYFP)). Heme binding induces dimerization, increasing FRET efficiency between ECFP and EYFP.^25^ In contrast, Hanna *et al.*^28^ developed a fusion of heme-binding cytochrome *b*_562_ with the fluorescent proteins EGFP and Katushka 2 (mKATE2),^28^ that show respectively heme-sensitive and heme-insensitive fluorescence, and therefore provide a ratiometric excitation–emission probe. Although these heme detection platforms^27^–^29^ are designed specifically for live cell measurements, the requirement for expression of the large protein probes encoded on plasmids does represent a distinct drawback, as it is unclear what effect this genetic manipulation may have on cellular processes and how expression of these heme-binding proteins may affect heme homeostasis and alter the levels of free heme. In light of this, we report here a new and much simpler design approach for the development of a heme probe, starting from a short synthetic peptide derived from a natural heme-binding protein. Fluorescence of an incorporated non-natural amino acid is quenched upon heme binding, allowing use of the sensor to detect heme in a biological medium.

A variety of short Cys-containing peptides from heme-binding proteins have been shown to bind heme independently with μM affinities.^30^–^32^ Bach1 contains four Cys-containing heme regulatory motifs, and these Cys-Pro units are all implicated in heme binding.^33^ Nine-residue peptides (**CP3–CP6**) from the Bach1 sequence around these motifs were synthesised along with a control (**AP3**) with the Cys residue mutated to Ala, and a Cys-Pro-containing peptide derived from the protein IRP2 whose interaction with heme has been previously characterised.^30^ The peptides were synthesised by 9-fluorenylmethoxycarbonyl (Fmoc) solid phase peptide synthesis (SPPS) (Scheme 1) on Rink amide (**CP4**, **CP4**, **CP6**, **AP3**) or Wang resin (**IRP2**, **CP5**).

**Scheme 1:**
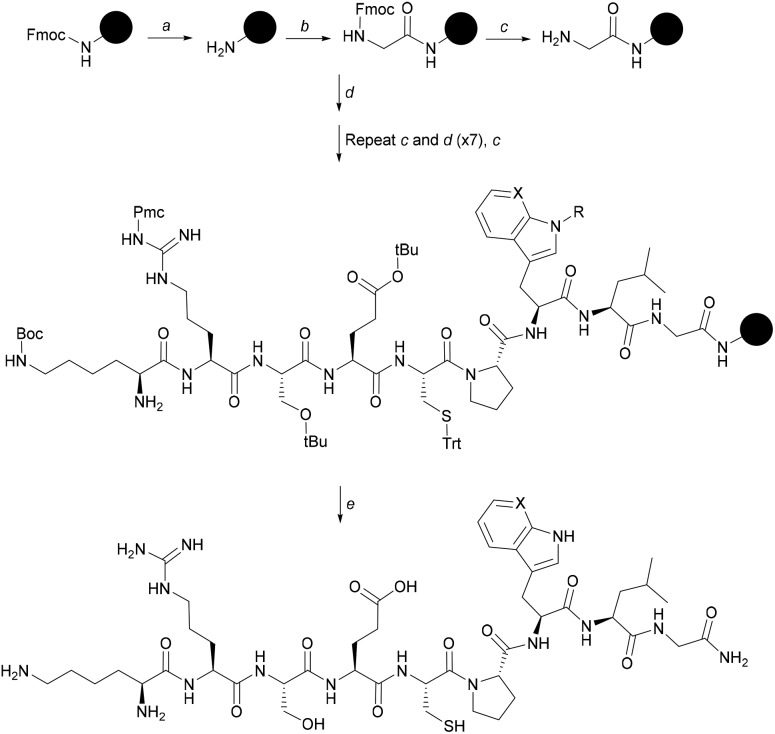
Synthesis of **CP3**, representative of other CP peptides. Reagents and conditions: a. Piperidine/DMF (1 : 5 v/v), 2 + 5 min b. Fmoc-Gly-OH, DIEA, PyBOP, DMF, RT, 1 h; c. piperidine/DMF (1 : 4 v/v), 5 + 10 min; d. Fmoc SPPS: Fmoc-AA-OH, PyBOP, DIEA, DMF; e. TFA/TIS/H_2_O/EDT (92.5 : 2.5 : 2.5 : 2.5, v/v/v/v), 3 h. For **CP3** (Trp-containing), X = CH and R = Boc. For **CP3[7azaW]**, X = N and R = H.

The interaction of the CP peptides with hemin (Fe(iii) heme) was investigated by UV-Vis spectroscopy. Kühl *et al.* have previously reported four different binding patterns for Cys-containing peptides with hemin^30^ where the form of the UV-Vis difference spectra obtained may be correlated with the peptide sequence and the coordination of iron.^34^ The spectra obtained for **CP4–6** (Fig. 1) correspond to one of the types identified by Kühl, with two absorption maxima at 367 nm (Near UV) and *ca.* 420 nm (Soret), which is observed for a number of peptides with a Pro residue following the heme-binding Cys, as in the CP peptides here. **CP3** however, seems to represent a new binding pattern with two minima at 340 nm and 397 nm. Like the peptides described previously,^30^**CP3–6** lack a negative amino acid on the C-terminal side of Cys, but uniquely **CP3** does contain a positively charged amino acid. As with many other heme-binding CP-sequences, the four Bach1-derived peptides have a hydrophobic or aromatic amino acid residue C-terminal to the Cys residue.^30^,^34^

**Fig. 1:**
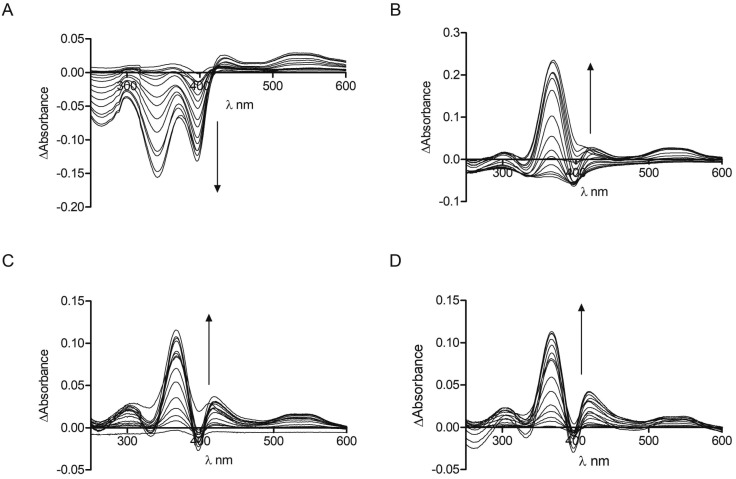
UV-Vis spectroscopy of CP peptides with hemin. Peptide concentration was constant (10 μM) whilst hemin was titrated (0.25 μM, 0.5 μM, 0.75 μM, 1 μM, 2 μM, 3 μM, 4 μM, 5 μM, 7.5 μM, 10 μM, 12.5 μM, 15 μM, 17.5 μM, 20 μM, 25 μM) in phosphate buffer (10 mM). After 2 min stirring, absorbance was read between 250 nm and 650 nm. (A) **CP3**. (B) **CP4**. (C) **CP5**. (D) **CP6**. Arrows denote increasing hemin concentration.

These data were used to calculate a *K*_d_ by fitting to the one-site binding equation (Table 1).^35^ The CP peptides tested here have *K*_d_ values ranging from 0.26 μM to 33.9 μM which are comparable to those reported for similar peptides by Kühl *et al.*^30^ The ΔAbsorbance data of **CP5** could not be fitted to the binding equation and a *K*_d_ value could not be obtained, which could be due to the presence of two Cys residues that allow more complicated peptide-heme structures to form. **CP5** also has two Pro residues which could contribute to conformations that do not favour strong heme binding compared to the other Bach1-derived peptides. As anticipated, very little heme binding was seen for **AP3** and again, the data could not be fitted to the one-site binding equation. The ΔAbsorbance data in this case were comparable to those seen for **CP3** with protoporphyrin IX (PpIX), the immediate heme precursor lacking an iron centre (see ESI†). This confirms the key roles of the Cys residue and iron in heme recognition and also provides an indication for the selectivity of **CP3** for heme over other intracellular biomolecules that might potentially cross-react with the probe.^25^

**Table 1:** Maximum wavelengths of CP peptides and the corresponding calculated dissociation constants. Data are means ± SEM

	Amino acid sequence	Near UV (nm)	Near UV *K*_d_ (μM)	Soret (nm)	Soret *K*_d_ (μM)
**CP3**	^431^KRSECPWLG^439^	340	—	397	1.7 ± 1.0
**CP4**	^457^SSVNCPFIS^465^	367	33.9 ± 11.1	420	—
**CP5**	^488^QQEPCPYAC^496^	367	—	419	—
**CP6**	^642^SAADCPLSF^650^	367	0.26 ± 0.24	421	—
**IRP2**	TPILCPFHL	367	0.89 ± 0.16	418	20.2 ± 4.6
**AP3**	KRSEAPWLG	347	—	420	—

Tryptophan is often the residue of choice in iFRET experiments to investigate ligand binding to proteins.^36^,^37^ Based on the UV-Vis data, we focused on the Bach1-derived peptide **CP3** with a Trp next to the key heme-binding CP motif. The principle UV absorption band of hemin overlaps with the emission of Trp in **CP3** at *ca.* 360 nm. Addition of hemin to **CP3** indeed reduced the fluorescence of the peptide in proportion to the amount of hemin present (Fig. 2) yielding a *K*_d_ for hemin binding of 0.44 ± 0.12 μM, comparable to the value obtained for **CP3** by the UV-Vis method (see Table 1).

**Fig. 2:**
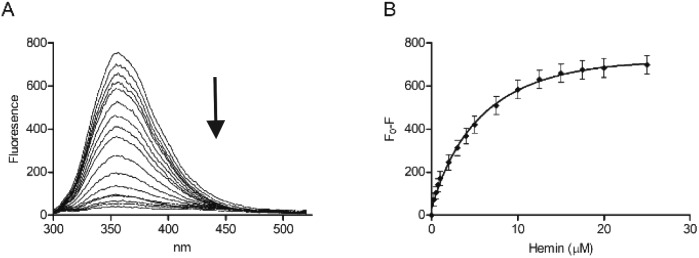
Fluorescence spectroscopy of **CP3** with hemin. Peptide concentration was constant (10 μM) whilst hemin was titrated (0.25 μM, 0.5 μM, 0.75 μM, 1 μM, 2 μM, 3 μM, 4 μM, 5 μM, 7.5 μM, 10 μM, 12.5 μM, 15 μM, 17.5 μM, 20 μM, 25 μM) in phosphate buffer (10 mM). After 2 min stirring, fluorescence was measured between 300 nm and 550 nm. Excitation wavelength was 280 nm; slit widths for excitation and emission were 5 nm. (A) Quenching of the fluorescence peak of **CP3** with addition of hemin. (B) *F*_0_ – *F* (fluorescence of peptide – fluorescence at each hemin concentration) for **CP3** with addition of hemin. Error bars show standard deviation, *n* = 4.

The modulation of tryptophan fluorescence in **CP3** upon hemin binding is promising for probe development and confirms that the position after the CP motif is close enough for energy transfer to occur between the bound hemin and the Trp residue. However, for heme sensing in cells it would be very difficult to quantify the Trp fluorescence of an added probe above that due to endogenous proteins. A fluorophore with a shifted fluorescence compared to Trp was therefore required to provide a readout that could more easily be distinguished from the background signal of cellular proteins.

A variety of tryptophan analogues are known that possess red-shifted fluorescence compared to the natural amino acid, allowing for their detection above the background Trp fluorescence of the cell.^38^–^40^ Among these, azatryptophans wherein one of the CH units of the indole ring is simply replaced with nitrogen^41^ are an attractive starting point as the minimal structural modification is not expected to significantly perturb binding of heme to a given peptide probe. 7-Azatryptophan (7-azaTrp) is readily available and has previously been incorporated into peptides by standard Fmoc SPPS and changes in fluorescence used to monitor binding to different substrates such as the protein thrombin^42^ or bacterium-like particles.^43^

A peptide with the sequence KRSECP[7azaW]LG was thus synthesised as before using a commercially available 7-azaTrp derivative (Scheme 1). Substitution of Trp with 7-azaTrp red-shifted the absorbance by 10 nm from 280 nm to 290 nm and the emission by 43 nm from 357 nm to 400 nm. For the new peptide, **CP3[7azaW]**, addition of hemin quenched the fluorescence maximum at 402 nm, and plotting *F*_0_ – *F* at the emission peak gave a very similar profile as for **CP3** with fluorescence quenching plateauing after 10 μM hemin (1 : 1 equivalents of peptide and hemin) (Fig. 3). Using these data to calculate a *K*_d_ yielded a value of 0.74 ± 0.42 μM, which is similar to the *K*_d_ found by fluorescence quenching for unmodified **CP3**.

**Fig. 3:**
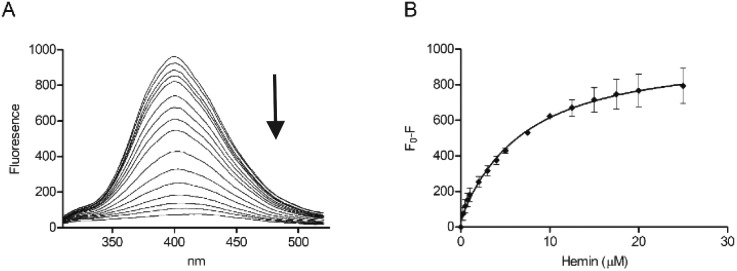
Fluorescence spectroscopy of **CP3[7azaW]** with hemin. Peptide concentration was constant (10 μM) whilst hemin was titrated as in Fig. 2 in phosphate buffer (10 mM). After 2 min stirring, fluorescence was measured between 300 nm and 550 nm. Excitation wavelength was 290 nm; slit widths for excitation and emission were 5 nm. (A) Quenching of the fluorescence peak of **CP3[7azaW]** with addition of hemin. (B) *F*_0_ – *F* (fluorescence of peptide – fluorescence at each hemin concentration) for **CP3[7azaW]** with addition of hemin. Error bars show standard deviation, *n* = 3.

As a proof of concept for the use of **CP3[7azaW]** and related peptides in heme determination in biological media, we tested its ability to detect changes in heme levels in FEK4 skin cell lysates, post-UVA irradiation (Fig. 4). The UVA component of solar radiation is a carcinogen and induces a local inflammatory response in the skin, and heme and HO-1 may contribute to these pathologies.^44^,^45^ It is therefore of great interest to be able to probe heme levels in skin cells after UVA exposure.

**Fig. 4:**
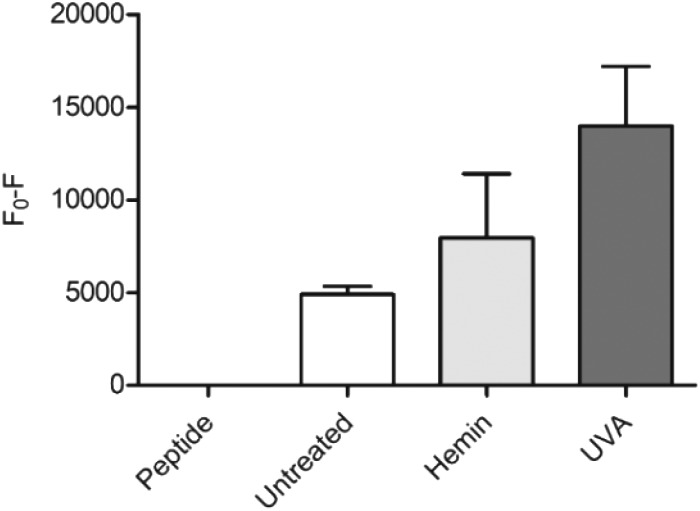
Detection of heme in FEK4 cell lysates with **CP3[7azaW]**. Cells were either untreated, treated with 10 μM hemin for 18 h, or exposed to 250 kJ m^–2^ UVA irradiation, and then lysed by sonication. **CP3[7azaW]** (10 μM) was then incubated with cell lysate containing 5 μg protein for 5 min. Fluorescence of peptide alone was designated *F*_0_. *n* = 4, error bars show SEM.

Quenching of the peptide fluorescence was seen when the cells were loaded with hemin compared to the untreated cells suggesting that the peptide was indeed detecting a change in heme levels. *F*_0_ – *F* was further increased by treatment with UVA irradiation compared to untreated cells (*p* = 0.06) consistent with the proposition that UVA irradiation directly increases free heme levels *via* degradation of hemoproteins.^45^

The applied dose of UVA is physiologically relevant, but relatively low, equivalent to only 45–60 minutes of sunlight exposure at midday in Southern Europe.^46^ A more substantial difference between the untreated and the UVA irradiated cells might therefore be expected with higher, non-physiological UVA doses. For the hemin-treated cells, the relatively low response may be attributable to increased expression of HO-1 in response to the added reagent, thus degrading much of the excess heme added. Notwithstanding this, in both scenarios, the modified **CP3** peptide could be used to demonstrate a perturbation in heme levels relative to the control. Importantly, the determination could easily be performed in a convenient microplate format.^25^,^26^

In conclusion, we have shown that a short peptide derived from a natural heme-binding protein can provide a simple starting point to develop a fluorescent probe for heme detection. The prototype molecule described herein could be used to detect changes in heme levels in a biological context, and has the great advantage that it can be readily further optimised by standard peptide chemistry to incorporate alternative reporting fluorescent amino acids, or to enhance its cell permeability and stability. This should provide a novel and flexible method to directly follow changes in heme levels in any number of cellular systems, without the need for genetic manipulation of the cells involved.

## Conflicts of interest

There are no conflicts to declare.

## Supplementary Material

Supplementary information
