# Supplementary material for: Development of a peptide-based fluorescent probe for biological heme monitoring
Source: Org Biomol Chem. 2018 Dec 18;17(3):467–71. doi: 10.1039/c8ob02290a (PMC6350759; doi:10.1039/c8ob02290a)
Supplement: Supplementary file 1 [file OB-017-C8OB02290A-s001.pdf]

Electronic Supplementary Material (ESI) for Organic & Biomolecular Chemistry.
This journal is © The Royal Society of Chemistry 2018

Development of a peptide-based fluorescent probe for biological heme monitoring

Laura D Newton,[a] Sofia I Pascu,[b] Rex M Tyrrell,[a] and Ian M Eggleston*[a]

[a] Department of Pharmacy and Pharmacology, University of Bath, Bath BA2 7AY, UK

[b] Department of Chemistry, University of Bath, Bath BA2 7AY, UK

Contents

S1. Materials and methods

S2. Characterisation of heme-binding peptides

S3. HPLC spectra of peptides

S4. Mass spectra of peptides

S5. UV-Vis spectroscopy of peptide **AP3** with hemin and peptide **CP3** with protoporphyin IX (PpIX)

S1. Materials and methods

Fmoc-protected amino acids were all obtained from NovaBiochem, with the exception of 7-azatryptophan (SigmaAldrich). Peptide grade DMF was purchased from Rathburn Chemicals. All other solvents were purchased from Fisher Scientific.

Solid Phase peptide synthesis was carried out on either Rink Amide MBHA resin (0.78 mmol/g) or Wang resin (0.9 mmol/g) using automated peptide synthesiser (Activotec P11). The peptides were synthesised using a standard Fmoc protocol with PyBOP as the coupling agent. Side chain deprotection and cleavage from the resin was with TFA/H2O/TIS/EDT (92.5/2.5/2.5/2.5, v/v/v/v) at room temperature for 3 h. The peptide was then precipitated in diethyl ether, centrifuged and washed with diethyl ethyl three times. Peptides were purified by semi-preparative reverse-phase HPLC using a Dionex HPLC system equipped with a Phenomenex Gemini 5 µm C-18 (250 × 10 41 mm) column with a flow rate of 2.5 mL/min. The gradient elution system was 0.1% TFA in water (mobile phase A) and 0.1% TFA in acetonitrile (mobile phase B). The peaks were detected at 214 nm, 220 nm, 254 nm and 280 nm. The gradient was T=0 min B=5%, T=30 min B=95%, T=45 min B=95%, T=45.1 min B=5%, T=52 B=5%. Purified peptides were lyophilised and stored at -20 °C.

Purity was confirmed by analytical RP-HPLC which was performed using a Dionex UltiMate 3000 HPLC system equipped with a Phenomenex Gemini 5 µm C-18 (150 × 4.6 mm) column with a flow rate of 1 mL/min. The gradient elution system was 0.1% TFA in water (mobile phase A) and 0.1% TFA in acetonitrile (mobile phase B). The peaks were detected at 214 nm, 220 nm, 254 nm and 280 nm. The gradient was T=0 min B=5%, T=10 min B= 95%, T=15 min B=95%, T=15.1 min B=5%, T=18 min B=5%.

Mass spectrometry: All peptides were characterised by electrospray mass spectrometry on either a Bruker microTOF LCS spectrometer with time of flight quantitation, or a Bruker MaXis HD ESI-QTOF coupled to a Thermo Scientific Dionex Ultra High Performance Liquid Chromatography (UHPLC) unit. Samples were prepared in acetonitrile or methanol.

Stock solutions of peptide were made at 10 mM in potassium phosphate buffer (PB) (10 mM, pH 7.0). For titration experiments, stock solutions of hemin were prepared at 5 mM in 30 mM NaOH in 10 mM PB. All stock solutions were stored at -20 °C and freshly thawed before use.

UV-Vis absorption spectroscopy: UV-Vis spectroscopy was performed using a Lambda 650 UV-Vis spectrophotometer (Perkin Elmer). Titrations were carried by a modification of the procedure of Kühl (Kühl *et al.* 2011) whereby increasing concentrations of hemin (or protoporphyrin IX) were added to 10 µM peptide in PB (10 mM, pH 7.0). The titrant solution was prepared at 500 µM in PB with 10 µM peptide. The peptide concentration was kept constant and the following titrant:peptide ratios were used 0.025:1.0, 0.05:1.0, 0.075:1.0, 0.1:1.0, 0.2:1.0, 0.3:1.0, 0.4:1.0, 0.5:1.0, 0.75:1, 1.0:1.0, 1.25:1.0, 1.5:1.0, 1.75:1.0, 2.0:1.0, 2.5:1.0

Fluorescence spectroscopy: Fluorescence spectroscopy was performed using a Lambda 55 Fluorescence spectrometer (Perkin Elmer). For titration experiments, increasing concentrations of hemin were added to 10 µM peptide in PB (10 mM, pH 7.0) as for the UV-visible experiments. The titrant solution was prepared at 500 µM in PB with 10 µM peptide. The peptide concentration was kept constant and the following titrant:peptide ratios were used: 0.025:1.0, 0.05:1.0, 0.075:1.0, 0.1:1.0, 0.2:1.0, 0.3:1.0, 0.4:1.0, 0.5:1.0, 0.75:1, 1.0:1.0, 1.25:1.0, 1.5:1.0, 1.75:1.0, 2.0:1.0, 2.5:1.0.

Cell lysate hemin detection: FEK-4 human skin fibroblasts were cultured in Eagle's Minimum Essential Medium with Earle's salts and sodium bicarbonate (Sigma), supplemented with 2 mM L-glutamine (Invitrogen), 50 U/mL penicillin (Invitrogen) and 50 µg/mL streptomycin and 15% FBS (Sigma). Cells were maintained at 37 °C under 5% CO_2 in a humidified incubator.

Cells were treated with hemin (10 µM) in growth media for 18 h, protected from light. Cells were treated with a UVA dose of 250 kJ/m^2 using a broad-spectrum 4 kW UVA lamp (Sellas, Germany). Irradiation was in the dark at 25 °C. Cells were lysed in lysis buffer (KH_2PO4, 20 mM; ethylene-diaminetetraacetic acid (EDTA), 0.5 mM; PMSF, 0.1%; with a complete Mini EDTA-free protease inhibitor cocktail tablet (Roche)), by sonication for 14 s on ice (Rapidis 300, Ultrasonics, UK). Quantification of cellular protein level was with the Pierce BCA Protein Assay (ThermoFisher Scientific) according to manufacturer's instructions. Cell lysate containing 5 µg of protein was incubated with fluorescent peptide (10 µM) in lysis buffer for 5 min on ice. Fluorescence was measured using a Clariostar microplate reader (BMG Labtech).

S2. Characterisation of heme-binding peptides

CP3 KRSECPWLG Obtained: 60.2 mg, 28%. HPLC: R_{t-} 5.37 min. [Found (ESI+) 1074.5593 $[M+H]^+$, $C_{47}H_{75}N_{15}O_{12}S$ requires 1074.5513].

CP4 SSVNCPFIS Obtained: 46.2 mg, 35%. HPLC: R_{t-} 5.82 min. [Found (ESI+) 952.4618 $[M+H]^+$, $C_{41}H_{65}N_{11}O_{13}S$ requires 952.4557].

CP5 QQEPCPYAC Obtained: 32.5 mg, 67%. HPLC R_{t-} 5.24 min. [Found (ESI+) 1038.4072 $[M+H]^+$, $C_{43}H_{63}N_{11}O_{15}S$ requires 1038.4019].

CP6 SAADCPLSF Obtained: 53.6 mg, 36%. HPLC: R_{t-} 5.78 min. [Found (ESI+) 909.4306 $[M+H]^+$, $C_{39}H_{59}N_{9}O_{14}S$ requires 909.4135].

CP-IRP2 TPILCPFHL Obtained: 61.5 mg, 43%. HPLC: R_{t-} 6.61 min. [Found (ESI+) 1040.5574 $[M+H]^+$, $C_{50}H_{77}N_{11}O_{11}S$ requires 1040.5597].

AP3 KRSEAPWLG Obtained: 46.0 mg, 44%. HPLC: R_{t-} 5.24 min. [Found (ESI+) 1042.7592 $[M+H]^+$, $C_{47}H_{75}N_{15}O_{12}$ requires 1042.5792].

CP3[7azaW] KRSECP[7azaW]LG Obtained: 75.0 mg, 70.6%. HPLC: R_{t-} 4.48 min. [Found (ESI+) 1075.5450 $[M+H]^+$, $C_{46}H_{74}N_{16}O_{12}S$ requires 1075.5466].

S3. HPLC spectra of heme-binding peptides

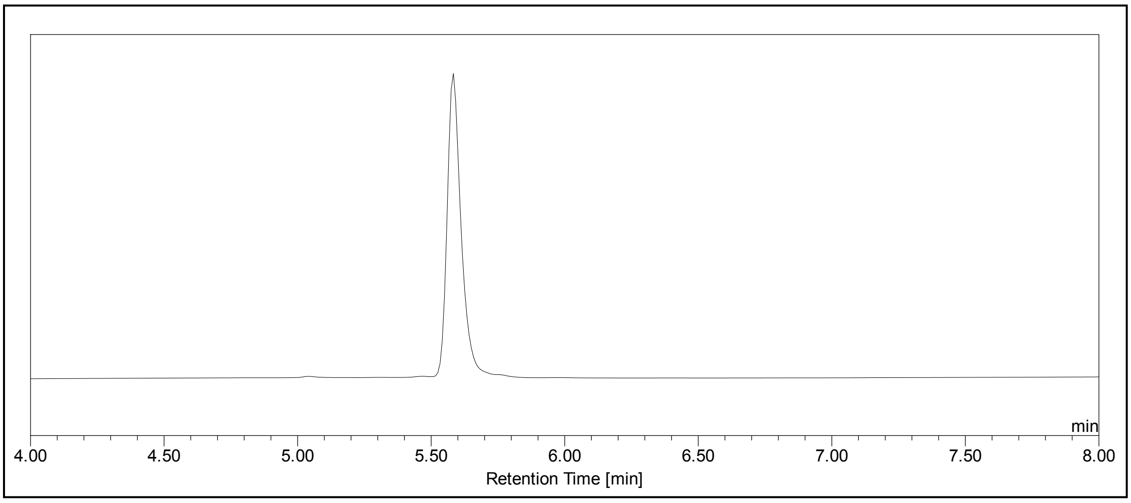

Figure S1. HPLC profile of **CP3**.

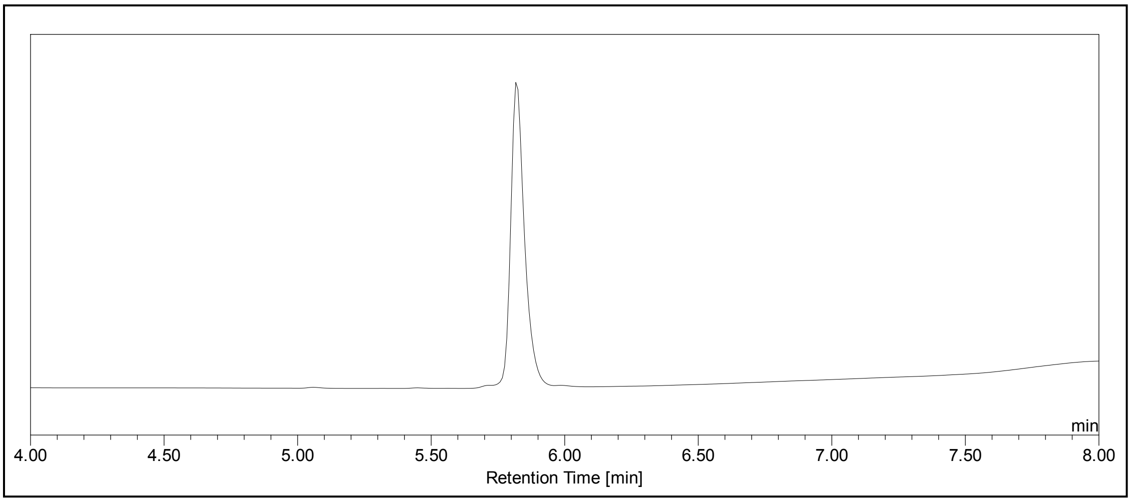

Figure S2. HPLC profile of **CP4**.

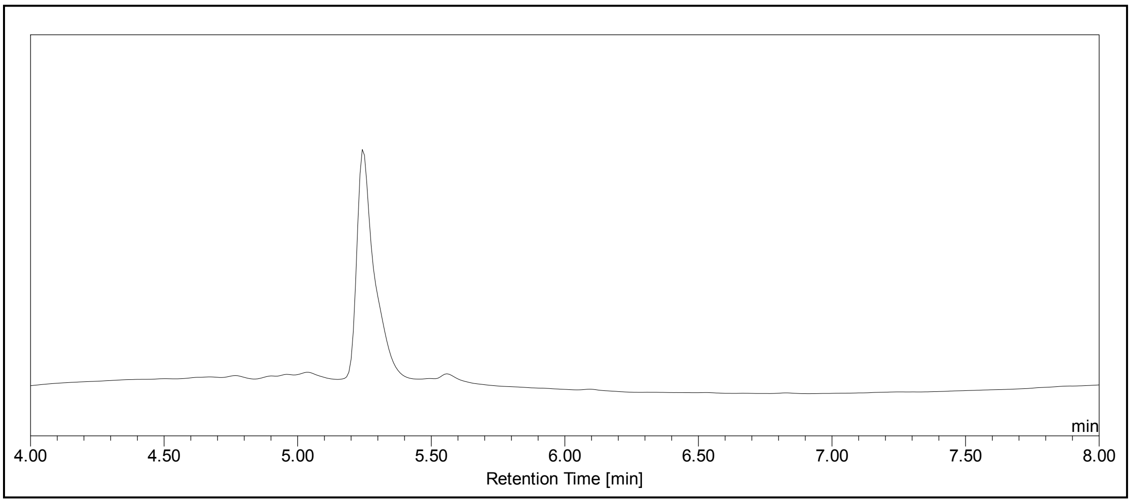

Figure S3. HPLC profile of **CP5**.

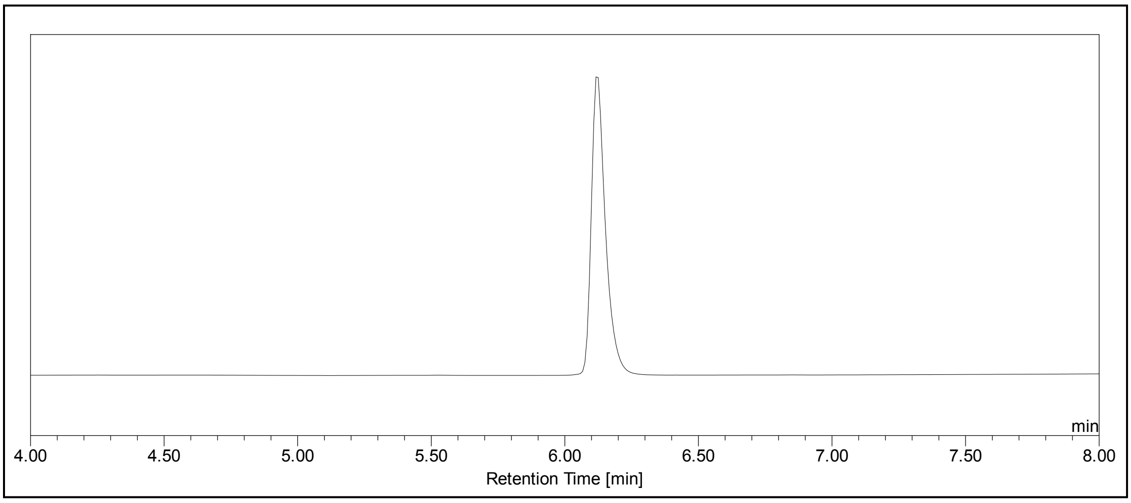

Figure S4. HPLC profile of **CP6**.

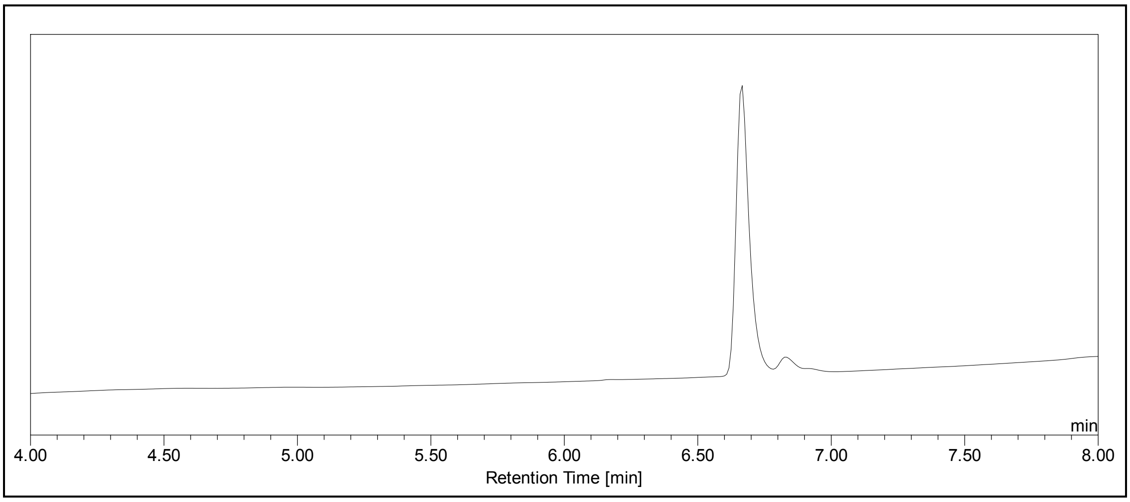

Figure S5. HPLC profile of **IRP2**.

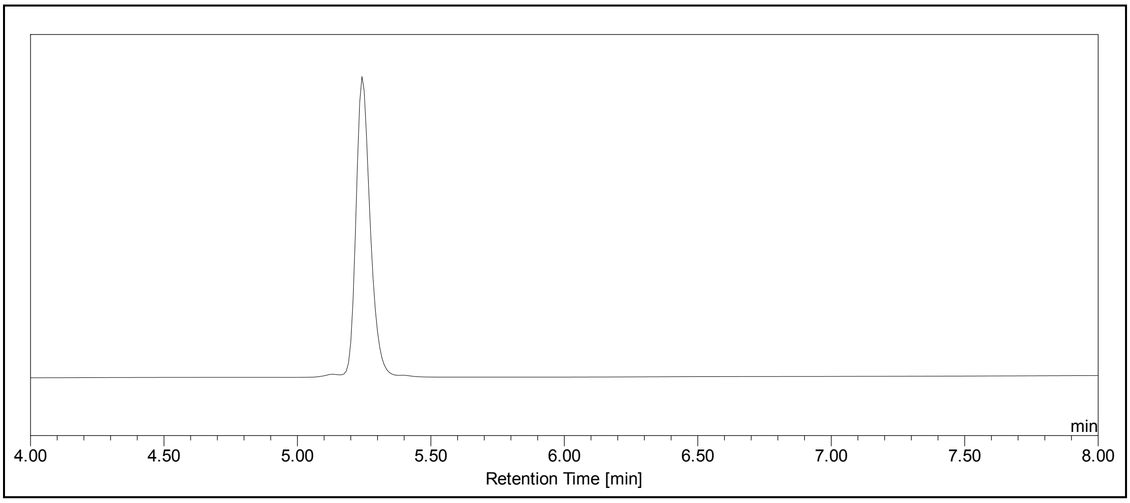

Figure S6. HPLC profile of **AP3**.

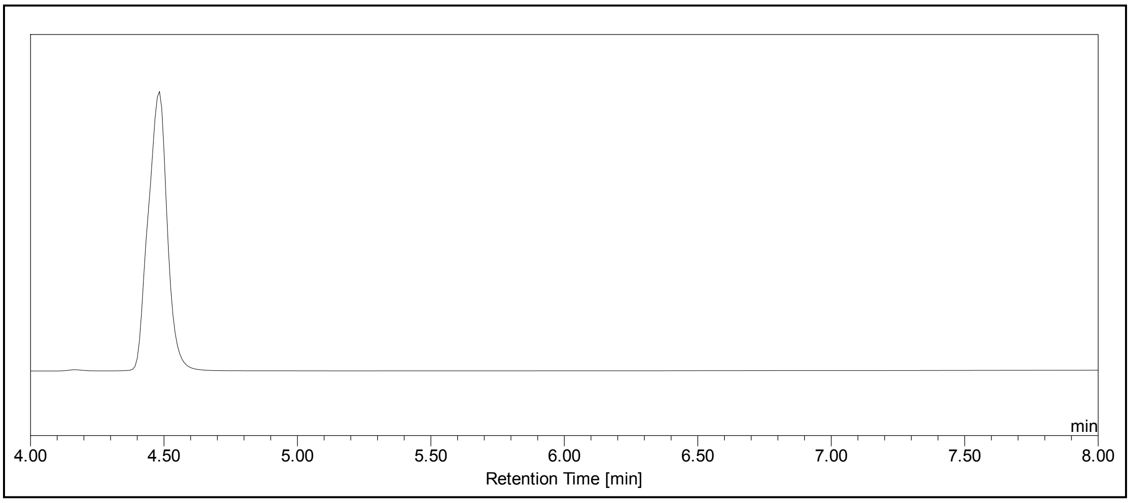

Figure S7. HPLC profile of **CP3[7azaW]**.

S4. Mass spectra of heme-binding peptides

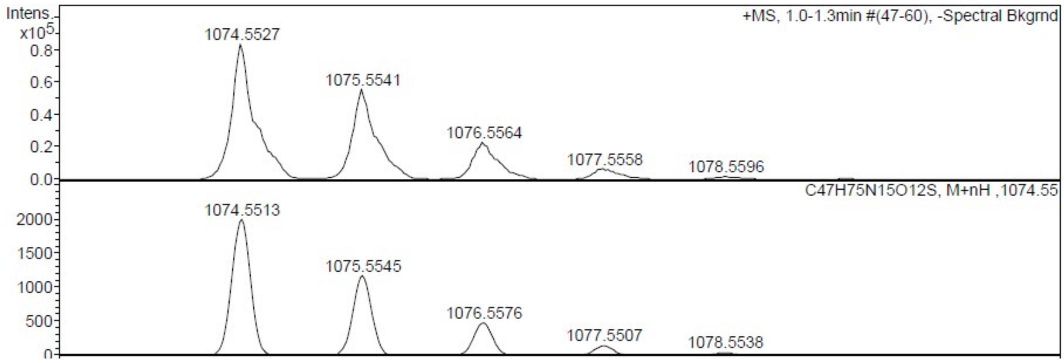

Figure S8. ESI mass spectrum of **CP3**.

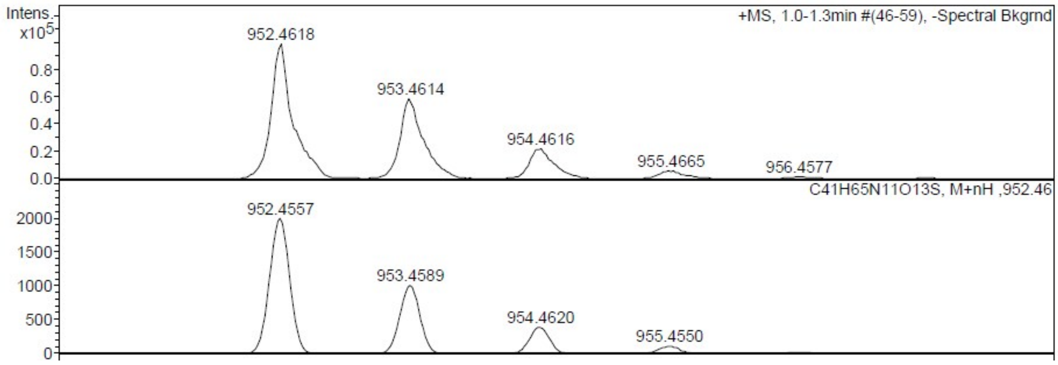

Figure S9. ESI mass spectrum of **CP4**.

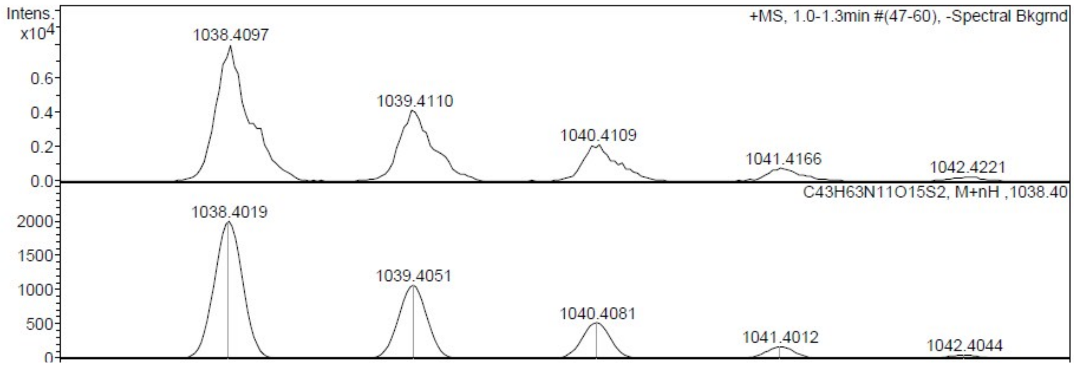

Figure S10. ESI mass spectrum of **CP5**.

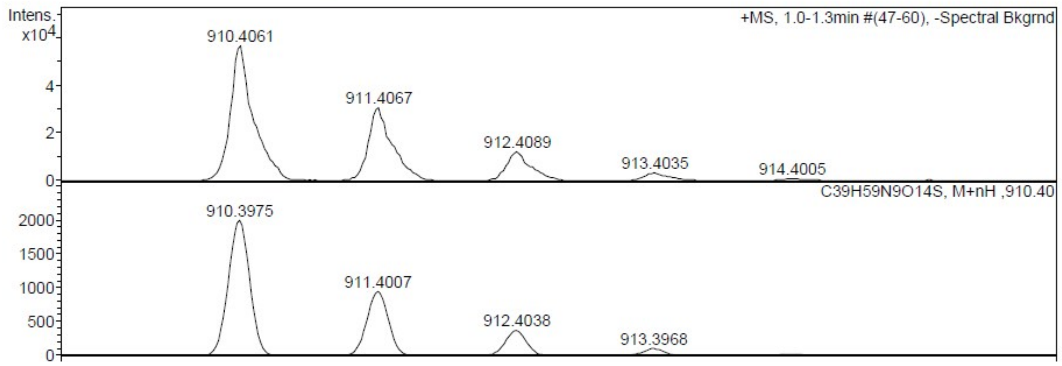

Figure S11. ESI mass spectrum of **CP6**.

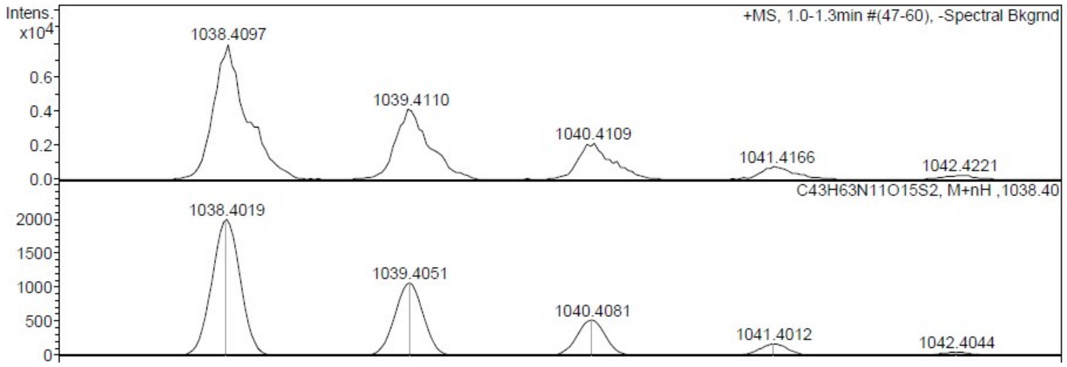

Figure S12. ESI mass spectrum of **IRP2**.

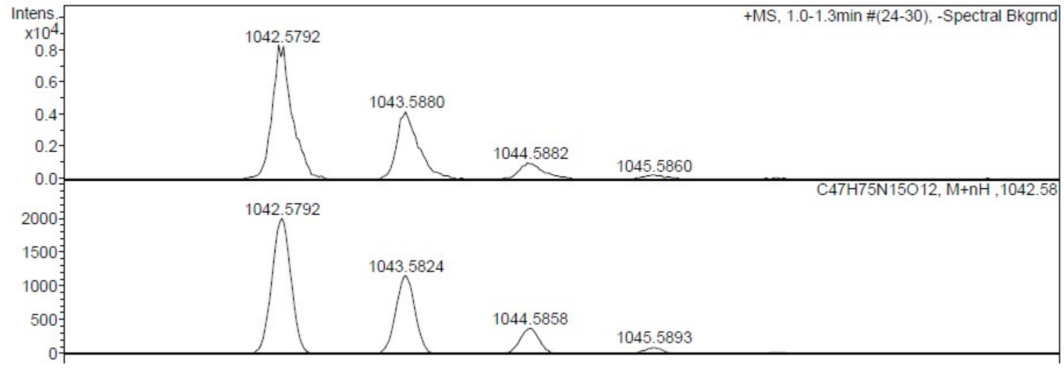

Figure S13. ESI mass spectrum of **AP3**.

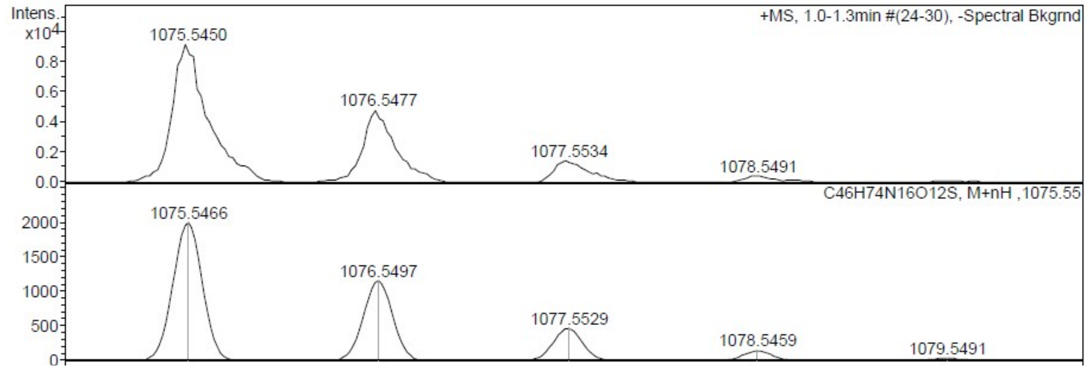

Figure S14. ESI mass spectrum of **CP3[7azaW]**.

S5. UV-Vis spectroscopy of peptides AP3 with hemin and CP3 with protoporphyrin IX (PpIX)

A

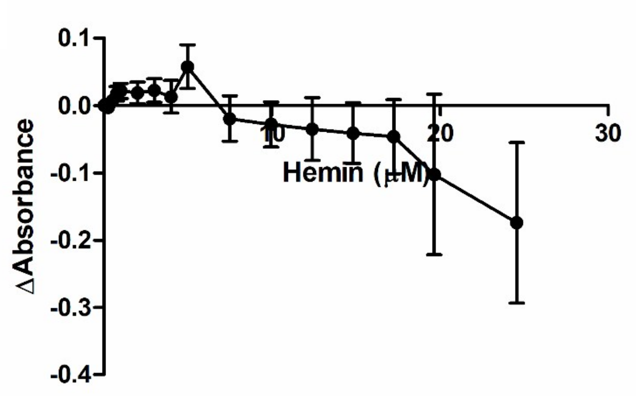

B

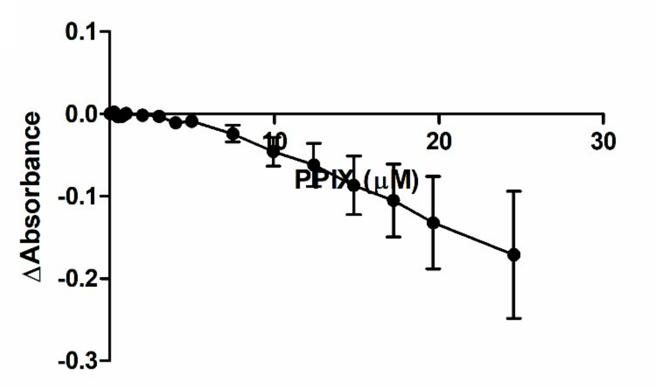

Figure S15. UV-Vis spectroscopy of **AP3** with hemin and **CP3** with protoporphyrin IX (PpIX). A) ΔAbsorbance of **AP3** with hemin at 347 nm against hemin concentration. B) ΔAbsorbance of **CP3** with PpIX at 342 nm against PpIX concentration. Peptide concentration was constant (10 µM) whilst hemin or PpIX was titrated (0.25 µM, 0.5 µM, 0.75 µM, 1 µM, 2 µM, 3 µM, 4 µM, 5 µM, 7.5 µM, 10 µM, 12.5 µM, 15 µM, 17.5 µM, 20 µM, 25 µM) in phosphate buffer (10 mM). After 2 min stirring the absorbance was read between 250 nm and 650 nm. Error bars show the standard deviation (n=3). In both cases, very little binding is evident until a concentration of 10 µM hemin or PpIX is reached (equivalent to a 1:1 ratio of peptide:hemin/PpIX).
